# An Ontology-Based, Mobile-Optimized System for Pharmacogenomic Decision Support at the Point-of-Care

**DOI:** 10.1371/journal.pone.0093769

**Published:** 2014-05-02

**Authors:** Jose Antonio Miñarro-Giménez, Kathrin Blagec, Richard D. Boyce, Klaus-Peter Adlassnig, Matthias Samwald

**Affiliations:** 1 Section for Medical Expert and Knowledge-Based Systems, Center for Medical Statistics, Informatics, and Intelligent Systems, Medical University of Vienna, Vienna, Austria; 2 Department of Biomedical Informatics; University of Pittsburgh, Pittsburgh, Pennsylvania, United States of America; 3 Medexter Healthcare GmbH, Vienna, Austria; The Cochrane Collaboration, Germany

## Abstract

**Background:**

The development of genotyping and genetic sequencing techniques and their evolution towards low costs and quick turnaround have encouraged a wide range of applications. One of the most promising applications is pharmacogenomics, where genetic profiles are used to predict the most suitable drugs and drug dosages for the individual patient. This approach aims to ensure appropriate medical treatment and avoid, or properly manage, undesired side effects.

**Results:**

We developed the Medicine Safety Code (MSC) service, a novel pharmacogenomics decision support system, to provide physicians and patients with the ability to represent pharmacogenomic data in computable form and to provide pharmacogenomic guidance at the point-of-care. Pharmacogenomic data of individual patients are encoded as Quick Response (QR) codes and can be decoded and interpreted with common mobile devices without requiring a centralized repository for storing genetic patient data. In this paper, we present the first fully functional release of this system and describe its architecture, which utilizes Web Ontology Language 2 (OWL 2) ontologies to formalize pharmacogenomic knowledge and to provide clinical decision support functionalities.

**Conclusions:**

The MSC system provides a novel approach for enabling the implementation of personalized medicine in clinical routine.

## Introduction

The goal of personalized medicine is to better tailor healthcare processes to the individual needs of patients. The use of genetic test results and other individual molecular markers is one of the most important means for achieving this goal [1]. While the range and quantity of genomic information relevant to drug therapy is growing rapidly, there are several challenges that need to be addressed in order for clinicians to apply genomic information to optimize therapy for their patients [2], such as:

- Processing complex genomic data generated by new sequencing technologies
- Identifying the effects of genomic variation on patient outcomes
- Applying these findings in order to improve medical practice

Pharmacogenomics is the study of how variability in drug response may correlate with the presence of certain sets of genetic variants within an individual or across a population [3]. It promotes the development of targeted therapies based on individual genetic variants and is one of the most promising facets of the personalized medicine research programme [4].

In recent years, pharmacogenomic studies have led to the discovery of a large number of genetic variants that correspond to drug response [5]. However, the limited pharmacogenomics training of prescribing and consulting clinicians [6], [7] and the growth of genetic knowledge bases hinder the inclusion of personalized medicine in clinical practice. The development of clinical decision support (CDS) systems for genetically guided personalized medicine has become an essential tool for anchoring pharmacogenomics in clinical routine [8].

Several systems that implement pharmacogenomic CDS in local institutions or regional infrastructures have been described. For example, Swen *et al.* reported on procedures followed to implement pharmacogenomic decision support rules in a nation-wide computerized drug prescription system in the Netherlands [9]. Pulley *et al.* reported encouraging results about using pharmacogenomic CDS for anticoagulant therapy at the Vanderbilt University Medical Center [10]. Lærum *et al.* recently reported good feedback from clinicians when testing a prototype of a pharmacogenomic decision support application for immunosuppressant dosing [11]. Kawamoto *et al.* provide a comprehensive review of clinical decision support systems for genome-based personalized medicine [8].

The Medicine Safety Code (MSC) system we present here provides a web-based, mobile-optimized CDS system aimed at facilitating personalized medicine and clinical pharmacogenomics across different institutions and regional infrastructures. Its goal is to support physicians with customized drug dosage recommendations and other treatment recommendations based on the genetic profiles of individual patients.

To achieve this goal, the MSC system needs to:

- Be able to parse genetic data to identify relevant genetic variations.
- Formally represent knowledge of the pharmacogenomics domain.
- Facilitate access to the inferred genetic markers and drug dosage recommendations.
- Address security and privacy issues related to the processing of personal health information.
- Be efficient enough to be deployed in routine medical care.

The current version of the MSC system is based on an early prototype first presented in a prior manuscript [12]. This prototype introduced the idea of encoding data on genetic polymorphisms in a two-dimensional (2D) barcode and offering a means to decode and interpret the data using common mobile devices. In this paper, we present the technical aspects of the first release of the full system, which is now based on ontological reasoning.

We chose to base our system on formal ontologies, semantic technologies and automated reasoning for implementing pharmacogenomic knowledge representation, quality assurance and clinical decision support. The rationale for choosing this approach was that in order to make effective use of pharmacogenomic biomarkers in routine medical care and clinical trials, the potentially large and complex data yielded by genotyping need to be reduced to more manageable, higher-level characteristics such as alleles, haplotypes, phenotypes or other classifications that can help to predict drug response. These higher-level classifications need to be clearly defined in order to avoid errors and inconsistencies in downstream clinical applications. This is a source of potential ambiguity and complexity, making it difficult to create reliable information technology systems for enabling clinical pharmacogenomics. We found formal ontologies to be a very good match to this problem domain.

In particular, we developed an OWL 2 ontology spanning from basic genetic markers to inferred treatment recommendations within a single, coherent model. This ontology contains a concise logical formalization of clinical pharmacogenomic definitions and rules. We use automated OWL 2 reasoning to detect potential errors in our knowledge base as well as to implement the clinical decision support algorithms for matching pharmacogenomic guidelines to individual genetic profiles.

## Materials and Methods

The MSC system provides two main functionalities: (1) Processing a patient's genotype profile to generate a two-dimensional MSC Quick Response (QR) code. This makes it possible for patients to carry their pharmacogenomic data with them so that the data are available at the point-of-care whenever needed. (2) Analyzing the genotype profile from a MSC QR code and providing decision support messages based on a patient's genotype. This enables medical professionals to use pharmacogenomic data contained in QR codes at the point-of-care.

The QR code specification [13] defines a standard 2D barcode representation for the visual codification of arbitrary data. QR codes have become very popular in media advertising campaigns and retailing for several reasons:

- They can be easily printed on all kinds of media.
- They can be quickly and robustly decoded even under suboptimal lighting conditions or viewing angles.
- Most current smartphones are shipped with pre-installed applets for decoding QR codes.
- They allow for embedding web hyperlinks.

The data capacity of QR codes mainly depends on the sizes (lines X columns) of the barcodes and their error correction levels. According to the QR code specification, codes can represent up to 23,648 binary digits. This technology, therefore, is ideal for systems that require a visual codification of data and a simple methodology for passing information to a software application. The MSC system uses QR code technology to represent the genetic variants of a patient and to provide this information to clinical decision support systems at the point-of-care in order to obtain appropriate drug recommendations.

**Figure 1:**
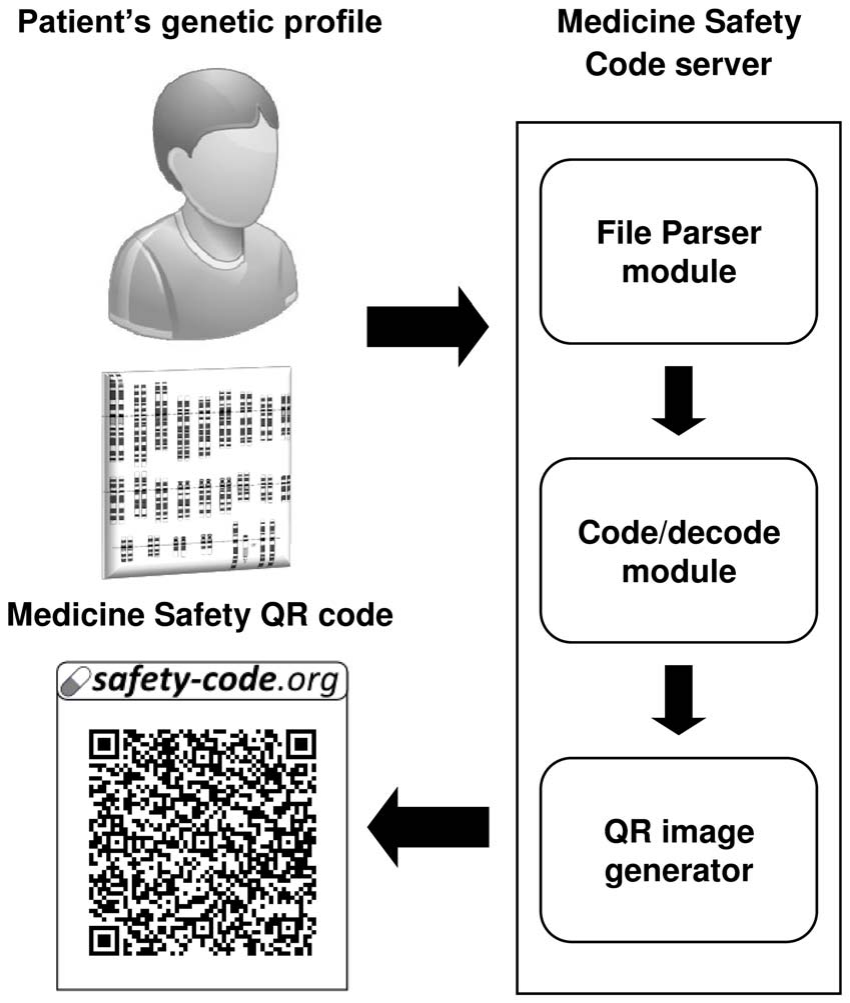
Processing a patient's genetic profile and obtaining the corresponding anonymous QR code.

The workflow of the genetic profile processing functionality is graphically described in Figure 1. A patient's anonymous genetic profile is uploaded to the web server and a suitable parser for the file format is chosen. Then, the corresponding parser module processes the genotype file and collects pharmacogenomic markers relevant for generating the MSC.

**Figure 2:**
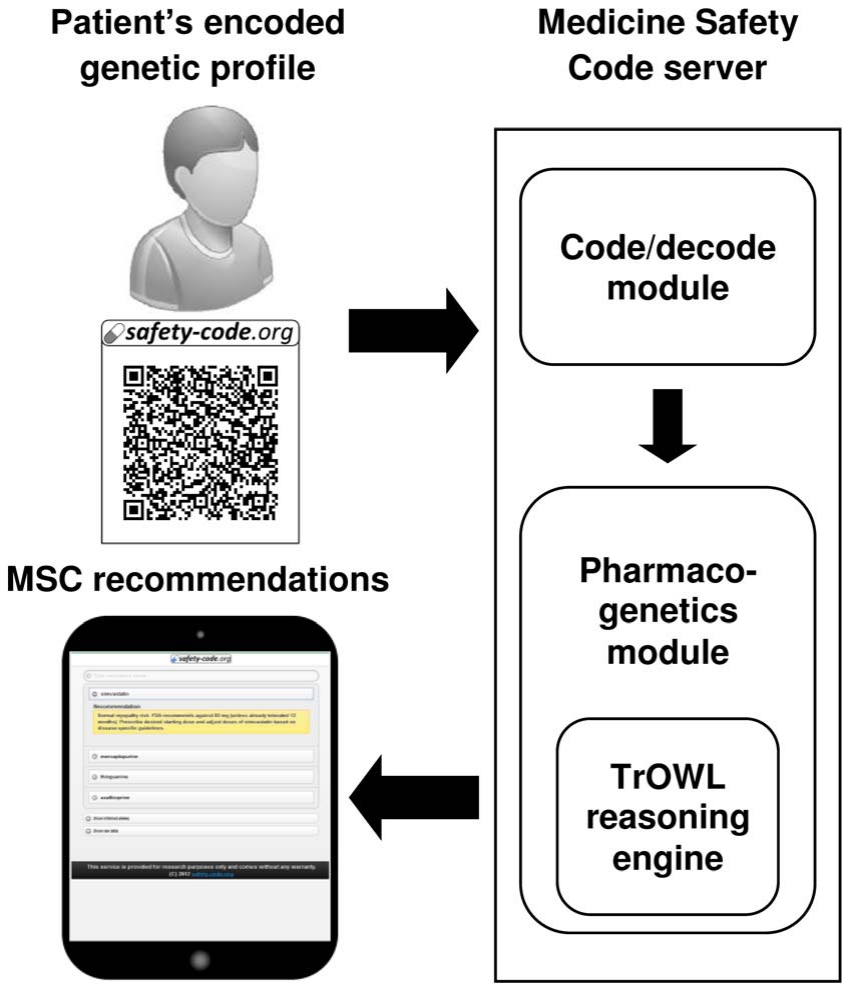
Reading and interpreting a genotype profile from a QR code.

The workflow of the second main functionality, the analysis of the patient's genetic profile, is presented in Figure 2. The MSC server takes as input the resulting QR barcode from Figure 1, runs a reasoner to infer matching CDS recommendations, and displays the matching CDS recommendations as an interactive HTML page. The components of the system are described in more detail below.

**Figure 3:**
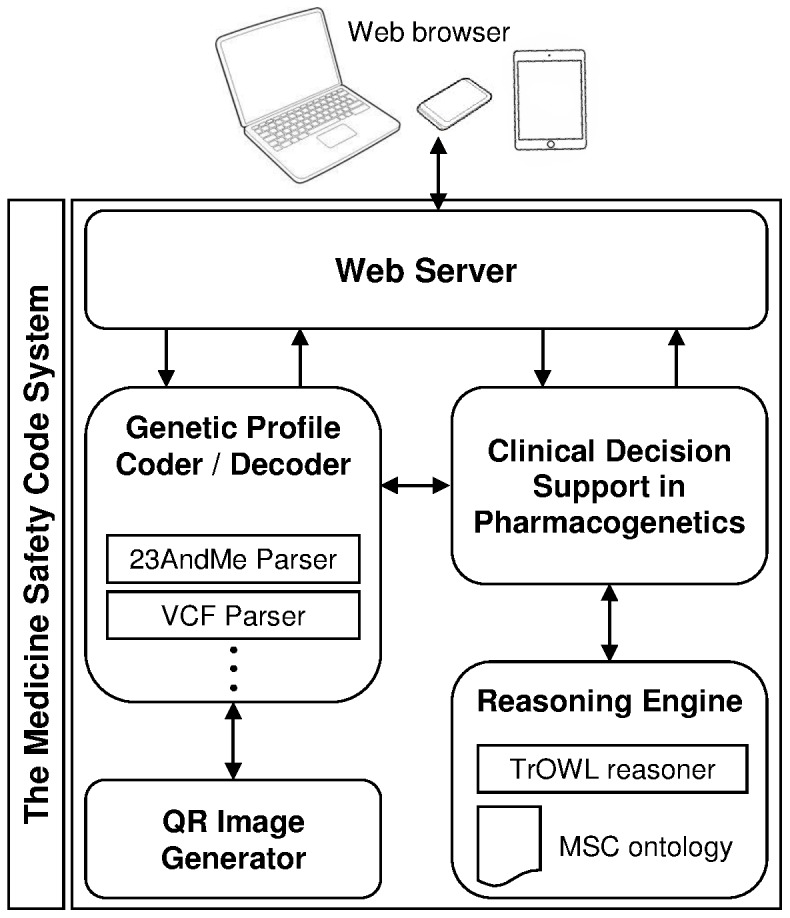
MSC system modules and their interactions.

The MSC system is based on Java technologies and on the model-view-controller software architecture. The user interface is based on the JQuery Mobile framework [14] to facilitate access from a wide variety of devices, including smart phones and tablets. JQuery Mobile enables the development of touch-optimized interfaces that automatically adapt to different screen sizes and device capabilities. Figure 3 shows a graphical representation of the module architecture.

### OWL 2 ontology

We created a list of 58 genes and 385 polymorphisms relevant to clinical pharmacogenomics by merging data from (a) the list of ‘very important pharmacogenes’ and their associated SNPs made available by the Pharmacogenomics Knowledge Base (PharmGKB) [15], (b) the PharmaADME core gene list [16], and (c) markers mentioned in FDA drug labels [17], excluding markers of somatic, non-inherited mutations. The following genes were represented in the ontology: ABCB1, ABCC2, ABCG2, ACE, ADRB1, ADRB2, AHR, ALOX5, BRCA1, COMT, CYP1A1, CYP1A2, CYP2A6, CYP2B6, CYP2C19, CYP2C8, CYP2C9, CYP2D6, CYP2J2, CYP3A4, CYP3A5, DPYD, DRD2, F5, G6PD, GSTM1, GSTP1, HLA-B*1502, HLA-B*5701, HMGCR, IL28B, KCNH2, KCNJ11, MTHFR, NAT1, NAT2, NQO1, NR1I2, P2RY1, P2RY12, PTGIS, PTGS2, SCN5A, SLC15A2, SLC19A1, SLC22A1, SLC22A2, SLC22A6, SLCO1B1, SLCO1B3, SLCO2B1, SULT1A1, TPMT, TYMS, UGT1A1, UGT2B15, UGT2B7 and VKORC1.

Top level classes and relations were created manually using the Protege 4 ontology editing environment [18]. The remaining parts of the ontology were developed via automated scripts as described below. We used the dbSNP batch query interface to download the dbSNP records for each of the 385 genetic markers.

We created scripts to parse PharmGKB haplotype/allele tables and to create OWL axioms representing the definitions in these tables. Since haplotypes are identified by sets of “tag” SNP variants [19], we formalized these as ‘necessary and sufficient’ conditions expressed as equivalentClass axioms, while all other known SNP variants were expressed as necessary conditions through subClassOf axioms. A simple example of OWL axioms created in this manner looks like this:

**Class**: ‘human with CYP2C9*3’
**EquivalentTo:**
  has **some** rs1057910_C
**SubClassOf:**
  has **some** ‘CYP2C9*3’,
  (has **some** rs1057910_C) **and**
  (has **some** rs1057911_A) **and**
  (has **some** rs1799853_C) **and**
  (has **some** rs2256871_A)

Decision support rules were created based on drug labels approved by the U.S. Food and Drug Administration (FDA), clinical guidelines of the clinical pharmacogenetics implementation consortium (CPIC) [20] and clinical guidelines of the Dutch pharmacogenomics working group [21]. The following code exemplifies an OWL representation of a dosing recommendation for the drug warfarin as described in an FDA product label (Coumadin, Bristol-Myers Squibb):

### 

**Class:** ‘human triggering CDS rule 9’

**Annotations:**

CDS_message “0.5–2 mg warfarin per day should be considered as a starting dose range for a patient with this genotype according to the warfarin drug label.”

**EquivalentTo:**

(has **some** ‘CYP2C9*1’) **and**

(has **some** ‘CYP2C9*3’) **and**

(has **exactly 2** rs9923231_T)

### Clinical Decision Support in Pharmacogenetics module

The core of the MSC system is the *Clinical Decision Support in Pharmacogenetics* module. This module implements the logic of the MSC system that provides drug recommendations based on patient genotype. The module accepts compressed data about the patient's genetic markers, decodes the data and infers the corresponding recommendations using the *Reasoning Engine* module. This module was implemented following the Singleton design pattern [22]. The Singleton minimizes memory consumption when offering common functions to several web requests and also contains a pre-computed load instance of the empty model that is used to reduce the processing time of each genotype analysis.

### The Reasoning Engine module

The *Reasoning Engine* module was implemented using Semantic Web technologies and based on the Genomic CDS ontology we developed recently [23], [24]. The Genomic CDS ontology is an OWL 2 DL ontology containing pharmacogenomic domain knowledge such as definitions of polymorphisms, alleles, phenotypes and treatment recommendations. When the genetic profile of a patient encoded in a QR barcode is submitted to the service, an OWL representation of the genetic traits of the patient is generated and combined with the domain knowledge in the Genomic CDS ontology. Then, an OWL 2 reasoner is used to infer matching alleles, phenotypes and treatment recommendations for the patient. We selected TrOWL 1.3 [25] as the main reasoner for the MSC service because of its significant advantages in reasoning performance and memory consumption compared to other OWL 2 DL reasoners we tested with the Genomic CDS ontology (paper under review).

**Figure 4:**
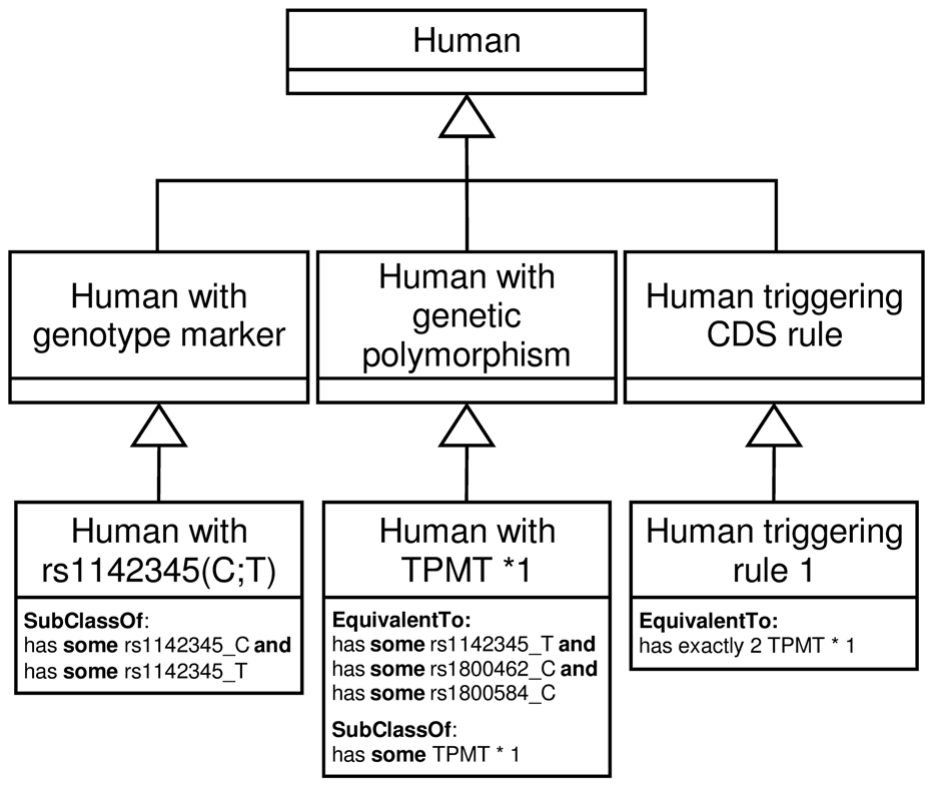
Excerpt of the OWL 2 ontology used for inferring matching polymorphisms and CDS rules from patient genotypes.

The Genomic CDS ontology conceptualizes the pharmacogenomics domain to represent the relations between humans, genetic markers (based on allele variations) and drug dosage recommendations. Figure 4 shows the excerpt of the Genomic CDS ontology that represents how genotype information (“*Human with genotype marker*” and “*Human with genetic polymorphism*” classes) is linked to patient (“Human” class) and drug recommendations (“*Human triggering CDS rule*” class). The ontology currently contains information on 1701 SNP variations, such as *rs1142345_C* which represent the variation with nucleotide C in the SNP *rs1142345* in human chromosome 6. There are also 556 alleles defined as a combination of SNP variations in the ontology, as well as 49 CDS recommendations for 6 different medicines.

In this module, the OWL API [26] is utilized to manage ontology access and reasoning capabilities provided by TrOWL. Matching CDS recommendations are inferred in three steps: first, the module is provided with the genetic profile of the patient; second, the information is represented in a newly created copy of the Genomic CDS ontology as an OWL individual of the class “human”; third, the TrOWL reasoner computes the individual's inferred classes (i.e., it realises the OWL individual) and finally, decision support messages attached to these inferred classes are forwarded to the *Clinical Decision Support in Pharmacogenetics* module to prepare the display of CDS recommendations.

### The genetic profile code/decode module

The web server allows users to choose different types of genotype file formats through the “Genetic Profile Encoder/Decoder” module.

The current version of the MSC system supports two widely used genetic file formats: the 23andMe format [27] and the Variant Call Format (VCF) [28]. These file formats contain textual representations of SNPs and the variants observed for each individual patient. The module is implemented using the Factory Method design pattern [22], which allows defining an interface to create objects of different types but making it easier to extend the system with new file parsers.

In the 23andMe file format, each line contains a pair of nucleotides associated with a particular SNP identified with an ‘rs’ number from the dbSNP database. It also provides information about the corresponding chromosome and position of the SNP. Files generated by the currently available 23andMe direct-to-consumer genetic test usually contain data about one million SNPs.

VCF files contain three main sections: meta-information, a header line and genotype information. The meta-information section describes the keys and the elements used in the section of genotype information. The header line indicates the order of tab-delimited data fields. The last section represents genotype information, with each line corresponding to a specific region in the genome. In most VCF files, only deviations from a specific human reference genome are listed, and missing information about a specific SNP is assumed to imply that the patient's genetic sequence does not differ from the reference sequence. Therefore, the MSC system assumes by default that the patient has the SNP variants in the reference sequence if they are missing from the VCF file.

### QR code generation, decoding and interpretation

The *QR code generator* module compresses data about a patient's pharmacogenomic markers to generate a corresponding MSC QR code. Internally, the genetic information of an individual is represented as a long binary number. Bits at specific positions in this binary number correspond to specific genetic polymorphisms (such as specific SNPs) in the Genomic CDS ontology. After this binary code has been generated from the patient's pharmacogenomic data, it is converted to a base 64 number to reduce its length and facilitate its transmission through QR codes. The compressed and encoded number is combined with the URL of MSC server (“http://safety-code.org/v0.2/<base_64_number>”). An example of a URL resulting from this process looks like this: http://safety-code.org/v0.2/QXGqrLF2h8xuqzIyCGJE2hzPzVzrND_q0vtKk2krxy0gQgDMlxWI0dzPwTq51w2UACs2nwZlF3QRxkv3uuuQtj4S55rDHGVU26maAZ203z-RCqhavsFv0a5uY1q770Su7_dg80000

To decode and extract the genetic markers from an MSC, the process described above is run in reverse: the base 64 number is obtained from the URL and transformed into a binary number; each combination of bits at specific positions in this binary number corresponds to a particular genetic polymorphism in the Genomic CDS ontology. The *reasoning engine* module creates an OWL individual of the class “human”, adds all genetic polymorphisms decoded from the URL to this OWL individual and obtains CDS recommendations that match the genetic profile of the patient. Finally, the recommendations are displayed as an HTML page and the MSC system releases the allocated resources, including the populated instance of the Genomic CDS ontology, for the next request.

## Results and Discussion

The MSC service is publicly accessible at http://safety-code.org/; the underlying source code and the most recent development version of the ontology are available from http://code.google.com/p/genomic-cds/ .

**Figure 5:**
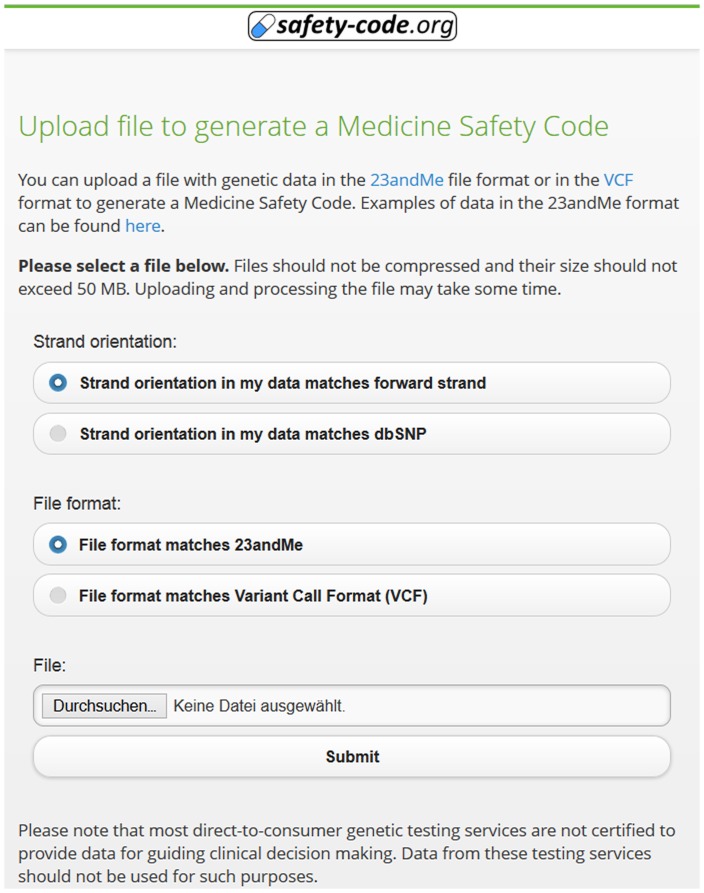
Interface to generate a QR code from a genetic profile in 23andMe or VCF format. For files in 23andMe format, the strand orientation of the genetic information can be chosen.

**Figure 6:**
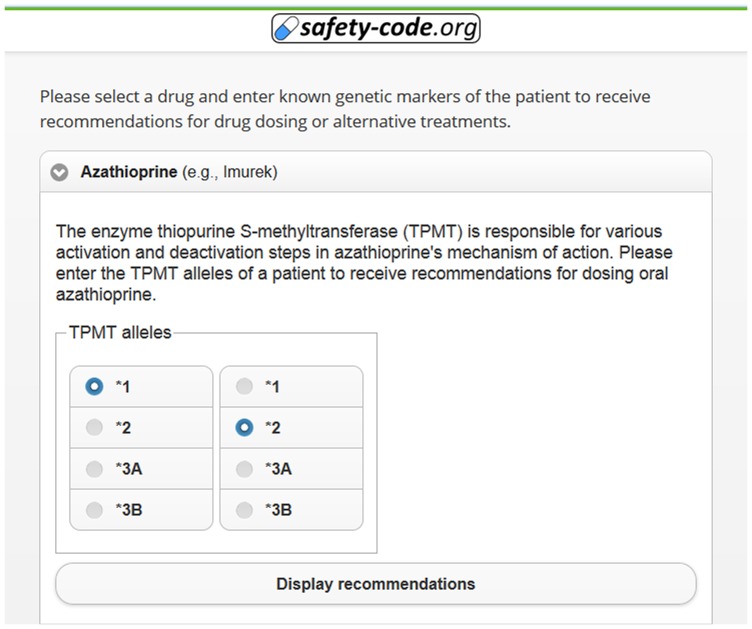
Interface for manual entry of genetic traits.

**Figure 7:**
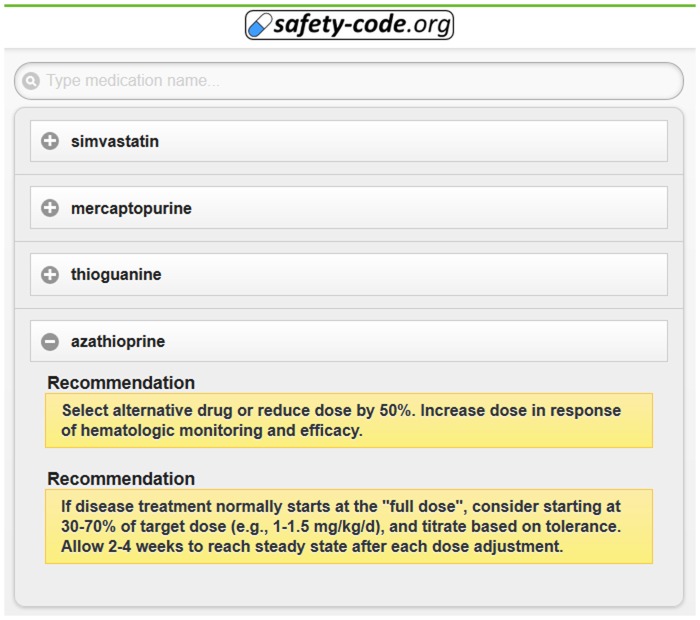
Example of simple pharmacogenomics-based treatment recommendations generated from a QR code. The current user interface displays basic recommendations, but future versions of the interface will also allow displaying further information – such as underlying mechanisms and evidence – when required.

The MSC system makes it possible to encode and compress 385 genetic polymorphisms in a two-dimensional barcode and provides the means to access the inferred drug recommendations using common mobile devices. The system showcases complex OWL 2 reasoning - based clinical decision support, has modest system requirements, and allows implementation of future extensions with little effort. Figure 5 demonstrates the interface for uploading a genetic profile to generate an MSC QR code. Some decision support algorithms can also be used through manual entry of relevant genetic markers (Figure 6). The interface for searching, browsing and displaying decision support messages after decoding an MSC QR code is exemplified in Figure 7.

### Limitations and advantages

A major advantage of the MSC system is that it can provide an open infrastructure for pharmacogenomic data sharing and decision support with very limited dedicated infrastructure. Other advantages are that sensitive genetic data are never explicitly associated with patient identities in the system, that central storage of genetic data is not necessary, and that local institutions can potentially create their own infrastructures completely independently.

The utilisation of an OWL 2 ontology to conceptualizes the pharmacogenomic domain allows for sophisticated consistency checking of the knowledge base, which helps to identify and correct possible errors in the complex definitions and rules encountered in pharmacogenomics. The Genomic CDS ontology can be sustainably kept up-to-date with current knowledge through semi-automated curation workflows based on data from relevant data sources such as dbSNP and PharmGKB.

Another advantage of the MSC system is its mobile-friendly interface, which automatically adapts to multiple mobile devices such as smartphones or tablets. Consequently, it allows more portability and flexibility than many established CDS systems, and the functionality provided by the system can be exploited in most health care settings. Mobile devices have been shown to help improve medical decision making in realistic clinical settings [29].

A major barrier to the practical utilisation of the MSC system is that pharmacogenomic testing is still not available to most patients. We are currently building partnerships with genetic testing providers to make genetic testing (and results in the form of MSCs) more broadly available in routine care.

Another major barrier is user acceptance. The MSC system aims to modify established workflows in the prescription and utilisation of medications, which is bound to meet resistance from both patients and medical practitioners.

According to an analysis of Kawamoto *et al.* [30] the success of CDS interventions is significantly correlated with four features:

1. Automatic provision of decision support as part of clinician workflow
2. Provision of recommendations rather than just assessments
3. Provision of decision support at the time and location of decision making
4. Decision support is computer-based (rather than based on non-electric systems)

While the MSC system exhibits features 2–4, we expect smooth integration of the system into existing workflows to be the most significant barrier towards user acceptance. The finding that system-initiated decision support systems are more effective than user-initiated systems is further substantiated by a study of Pearson *et al.* [31].

In this context, it is noteworthy that 2D barcodes are likely to become widely used for drug package tracking, tracing and verification. In several countries and some states of the US 2D barcodes on drug packages have recently become required by law [32], [33], and pharmacists and medical professionals are required to scan medications before they are dispensed. The main motivation behind these developments are improved logistics in the medication supply chain, substantially reduced incidence of errors in medication dispensing [34], and the need to counter the growing threat of counterfeit medications [35]. Albeit the majority of these developments use GS1 DataMatrix barcodes [36] or other types of barcodes instead of web-enabled QR codes, they will help establish the use of 2D barcodes as a common practice in medication handling and dispensing. This, in turn, might increase the chances of a successful introduction of the MSC into existing clinical workflows where quick scanning of 2D barcodes has become routine practice. Scanning of medication barcodes and MSCs could be integrated into a single app, which would also make it easier to correlate medications with the pharmaceutical compound that is about to be dispensed, as well as integrating drug-drug and drug-drug-gene interaction alerts in the case of dispensation of multiple medications.

Several studies on the effectiveness of CDS interventions in improving physician prescribing behaviour have been published over the last decade, but many studies also suffer from limited sample size and poor reporting of system features that might be associated with success [31], [37]. Significant problems related to system usability and information visualisation are commonly reported, and the adaptation of systems based on user feedback was demonstrated to improve user acceptance [38].

Both patients and medical practitioners might have doubts about the trustworthiness, security and privacy implications of the system. It is important that these issues are addressed with utmost care and transparency. The integration of major stakeholders from relevant areas of the health care space into development and dissemination will be essential for gaining acceptance. This includes clinicians, patient organisations, health insurance providers and pharmaceutical companies.

Previous work on guidelines for genome-guided therapy of psychiatric drugs has led to the recognition of the importance of considering pharmacogenomic information within the context of other influences on drug effectiveness and safety, such as exposure to potential drug-drug interactions, age-related clearance reductions, and co-morbidities [39]. Currently, the MSC is focused on providing decision support based on genetic markers, leaving the synthesis of pharmacogenomic findings with other patient parameters to the medical professional. It can be argued that this is a significant limitation of the system, and we plan to investigate how the CDS algorithms currently employed by the system could be enhanced to take other, non-genetic factors into account. However, the integration of further parameters into decision support algorithms might also make the validation of the system very difficult. In this regard, the current, narrow focus on matching patients to recommendations from existing clinical guidelines that have been vetted by expert committees might also be seen as a positive aspect of the system.

The amount of information that can be represented in a QR code of practical dimensions is limited. Currently, the MSC system captures data on 385 SNPs, but this number might be somewhat increased by using more sophisticated compression algorithms, or by encoding alleles and phenotypes instead of raw genetic markers such as SNPs.

On the current server hardware (Intel Xeon E3-1230, 3,30 GHz, 16 GB RAM) and using TrOWL version 1.3, the ontology-based reasoning engine takes 3 to 4 seconds to infer drug recommendations. This response time is not optimal for use in busy medical routine. The load on the OWL reasoner might further grow when the size and complexity of future versions of the Genomic CDS ontology increase. This issue is mitigated by the fact that the MSC system includes a caching functionality, so that results are available without delay for MSCs that have already been decoded and cached once. Furthermore, the performance of OWL reasoners – such as TrOWL – is still improving continuously.

### Related work

Several approaches towards representing pharmacogenomic data through ontologies are described in the literature. We analyzed these existing ontologies for their potential application to our use-case, but concluded that none of these existing resources could be adapted to our needs. The *SNP-Ontology* and the *Suggested Ontology for Pharmacogenomics (SO-PHARM)* [40] represent genetic variation using OWL description logic. These ontologies were formalized in OWL 1 and are unable to conveniently represent relevant knowledge captured in the ontology we created, which requires features that were introduced in OWL 2 (qualified cardinality restrictions). The SNP-Ontology and SO-PHARM are not actively maintained at the time of this writing. The *Clinical Bioinformatics Ontology (CBO*) contains information about pharmacogenomic variants [41]. However, it does not contain logical axioms for inferring alleles and matching guidelines through OWL reasoning and is not actively maintained at the time of this writing. *GENO* [42] is an ontological model of genotype information that aims to support data integration across model organism databases. GENO does not in itself represent important pharmacogenomic variants, furthermore, it cannot be used for the kind of reasoning and decision support enabled by the ontology we created.

There exists some previous work on CDS systems where decision support logic is partly or fully based on OWL reasoning and/or semantic technologies. One of the earliest examples is a system developed by Bouamrane *et al.* [43], which employs OWL reasoning together with other rule systems for preoperative assessment in order to identify potential risks and complications. A more recent example is the Lung Cancer Assistant system, which employs OWL reasoning for lung cancer treatment selection [44]. Douali *et al.* recently proposed a decision support system enabling personalized treatment recommendations based on Semantic Web tools and case-based fuzzy cognitive maps [45].

Alternative technologies that are competing with or are complementary to the MSC system include:

1. Electronic health record (EHR) systems that are able to capture pharmacogenomic data and provide decision support at the point-of-care. Unfortunately, we expect that capable EHR systems will remain unavailable or fragmented in most regions in the foreseeable future.
2. Rapid genetic testing technologies that can be used at the bedside and yield results in less than one hour. Currently, such tests focus only on single genes and re-use of results is difficult.

We assume that the Medicine Safety Code system can still add value in settings where such complementary technologies are deployed, e.g., EHR systems might not be available to all healthcare providers in a given region; the MSC decision support module can be plugged into EHR systems; results from rapid genetic tests can be captured as MSCs to save on assay costs in future patient encounters.

### Future work

We are currently working on updating the ontology used for decision support, adding new genetic markers and a large number of new decision support rules. We are also working on refining the detection and representation of other polymorphisms besides simple SNPs, such as insertions/deletions or short repeats. We will also work on representing information about drug allergies in the MSC.

Two important next steps need to be made towards practical application are setting up a quality control and validation process on one hand and conducting evaluations of the system in realistic clinical settings on the other hand.

For the current version of the system, the correctness of inferred recommendations was checked by ‘manually’ inspecting source data and clinical guidelines, and comparing the inferences and CDS messages made by the system with the source datasets and guidelines. This kind of validation is not sufficient for proving the system to be reliable enough for real clinical applications, and it is also not very efficient, i.e., these validations would need to be repeated with every new version of the system. We are therefore creating a collection of several genetic profiles that will act as ‘test cases’, and are setting up a system for automatically checking the inferences generated for these test cases by our system, i.e., we are working towards creating a more comprehensive unit testing framework.

Since our long-term goal is to employ the system (or some of its core components) for guiding medical decision making in clinical settings, we will also work towards setting up a comprehensive quality assurance system that is necessary for achieving certification of the system as a medical device (which is a legal requirement under EU and US legislation). The preparation for certification as a medical device will include the creation of a detailed risk analysis, setting up organisational workflows for responding to problems, and strategies for keeping the system up-to-date in light of new medical evidence.

The certification of the system as a medical device poses an exquisite challenge. Quality control and the continuous integration of new data into the system is facilitated by our reliance on (semi-)automated scripts for data import, as well as the use of ontologies and reasoners for knowledge base analysis and consistency checking. Still, a significant ongoing effort is required for meeting all criteria necessary for certification and maintaining a system that is sufficiently reliable for clinical application – requirements that are not easy to accommodate into classical academic research environments. We are currently working on acquiring additional funding for making sensitive modules of the system ready for certification as a medical device. We are also investigating monetization strategies to fund ongoing deployment and maintenance of the system while establishing the Medicine Safety Code as an open standard.

We are preparing for user tests with medical professionals and pharmacists in order to optimize the system for use in realistic clinical settings. Since the major focus of these first user evaluations is on the overall usability and acceptance of the system – and because the system will not be employed to guide medical treatment – these user tests can run in parallel with efforts for validating and certifying the decision support module.

## Conclusions

The unique approach of the MSC system reduces some of the patient confidentiality and technology acquisition barriers to the storage, processing and communication of sensitive personal data. The system could prove to be an enabling technology for the emerging era of personalized medicine.

## Availability and requirements

**Project name:** Medicine Safety Code web service

**Project home page:** https://code.google.com/p/genomic-cds/

**Operating system(s):** Platform independent

**Programming language:** Java

**License:** Dual licensing: AGPL 3.0 (for open-source projects), proprietary (for non-open-source projects)

**Any restrictions to use by non-academics:** Please contact the corresponding author if you plan to use this software or derivatives of this software for commercial purposes.
